# Deducing acidification rates based on short-term time series

**DOI:** 10.1038/srep11517

**Published:** 2015-07-06

**Authors:** Hon-Kit Lui, Chen-Tung Arthur Chen

**Affiliations:** 1 Department of Oceanography, National Sun Yat-Sen University, Kaohsiung 80424, Taiwan

## Abstract

We show that, statistically, the simple linear regression (SLR)-determined rate of temporal change in seawater pH (*β*_*pH*_), the so-called acidification rate, can be expressed as a linear combination of a constant (the estimated rate of temporal change in pH) and SLR-determined rates of temporal changes in other variables (deviation largely due to various sampling distributions), despite complications due to different observation durations and temporal sampling distributions. Observations show that five time series data sets worldwide, with observation times from 9 to 23 years, have yielded *β*_*pH*_ values that vary from 1.61 × 10^−3^ to −2.5 × 10^−3^ pH unit yr^−1^. After correcting for the deviation, these data now all yield an acidification rate similar to what is expected under the air-sea CO_2_ equilibrium (−1.6 × 10^−3^ ~ −1.8 × 10^−3^ pH unit yr^−1^). Although long-term time series stations may have evenly distributed datasets, shorter time series may suffer large errors which are correctable by this method.

Ocean acidification is an unavoidable consequence of increasing atmospheric CO_2_^1^,^2^,^3^,^4^,^5^. Understanding how fast the oceans acidify is critical to examining and predicting the impact of ocean acidification on the aquatic ecosystem. Invaluable decadal ocean time series measurements of carbonate chemistry parameters can now be used for this purpose. Observations show that surface ocean pH under *in situ* temperature (pH_insitu_) has strong seasonal variations, with a high pH_insitu_ in winter and a low pH_insitu_ in summer. Long-term decreasing trends are superimposed on the regular seasonal variations^6^,^7^,^8^.

To investigate how fast the oceans acidify, the widely used first-order simple linear regression (SLR) method has been used to model the long-term temporal changes in pH_insitu_ (*β*_*pH*_)^2^,^6^,^7^,^8^,^9^,^10^,^11^,^12^,^13^. The SLR model in general form is as follows,


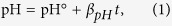


where pH° is the intercept, *β*_*pH*_ is the slope, and *t* is time in the pH_insitu_ vs. *t* plot.

The *β*_*pH*_, the so-called acidification rate, refers to an average rate of temporal change in pH_insitu_. Conventional wisdom has it that the longer the pH_insitu_ time series (e.g., over a decade) the better the estimation of the *β*_*pH*_. Bermuda Atlantic Time Series Study (BATS) and the Hawaii Ocean Time Series (HOT) (see Fig. 1 for the station locations) are two examples of long-term time series with even sampling distribution, and their *β*_*pH*_ values fairly reflected the acidification rates due to the increased atmospheric CO_2_ concentrations^6^,^7^. However, in a time series with short observation years (e.g., less than a decade), the small long-term temporal change in pH_insitu_ due to the increase in atmospheric CO_2_ could be easily masked by the strong seasonal variations, especially in the case of uneven sampling distribution. The pH_insitu_ and seawater temperature (*T*) time series at the European Station for Time Series in the Ocean Canary Islands (ESTOC) between 1995–2009 are shown as examples (Fig. 2). The raw data (blue cross), when fitted by the SLR, result in a negative *β*_*pH*_ of −1.84 ± 0.39 × 10^−3^ pH unit yr^−1^ and positive SLR-determined rate of temporal change in *T* (*β*_*T*_) of 0.023 ± 0.039 °C yr^−1^ (Fig. 2). The red circles represent an extreme example of uneven sampling distribution, with the sampling time gradually shifting from summer to winter. As a result, these data now would have a positive *β*_*pH*_ of 1.61 ± 0.37 × 10^−3^ pH unit yr^−1^ and a negative *β*_*T*_ of −0.273 ± 0.036 °C yr^−1^. Moreover, the slopes of the regression lines would increase with a shift in sampling time from summer to winter within a shorter observation period. This shows that *β*_*pH*_ is highly sensitive to the temporal distribution, and the duration, of time series data.

It has been suggested that the effect of seasonal variations on *β*_*pH*_ could largely be reduced when pH anomaly data (observed data minus seasonal average) are used^2^. However, this method may not be applicable to time series with short observation periods and uneven sampling distribution, which do not have sufficient data to generate effective seasonal means, resulting in errors. Furthermore, the reported low values of the coefficient of determination (R^2^), ranging from 0.09 to 0.55, indicate that these time series data were still poorly fitted by SLR lines^2^. Consequently, estimating *β*_*pH*_ using only *t* introduces a deviation due to the influence of strong temporal variations. As a result, it remains unclear whether the observed rates at which surface seawater pH_insitu_ changes differ are due to the changing carbonate chemistry of seawater at different time series stations or to deviation using SLR.

In this study, we propose a simple statistical method for reducing deviations of *β*_*pH*_ under the influence of strong temporal variations. We show that when pH is expressed as a linear function of *t* and the other variables, *β*_*pH*_ can be expressed as a linear combination of a constant (an estimated rate of temporal change in pH) and SLR-determined rates of temporal changes in the selected variables (deviation largely due to various sampling distributions under the strong seasonal pH variability), despite complications due to different observation durations and temporal sampling distributions. Using this method, we demonstrate that *β*_*pH*_ differs from the observations from five time series stations (the BATS, Carbon Retention in a Colored Ocean Project (CARIACO), ESTOC, HOT, and the South East Asia Time Series Study (SEATS, with bottom depth >3700m)) mainly due to deviations under various sampling distributions rather than changing seawater chemistry. Average summer and winter *β*_*pH*_ values from 31 stations along the 137°E repeated hydrographic line (137°E) likely contain the same deviation.

There are few reported acidification rates for the world’s oceans, as there are only a handful of stations with decadal time series of carbonate parameters. Time series studies with high temporal variations and few observation years can benefit from our approach to find the fair estimation in the rate of temporal change in pH_insitu_.

## Results

### Decomposition of *β*_*pH*_ and its physical meaning

Statistically, for any pH_insitu_ time series, *β*_*pH*_ is defined as follows^14^,


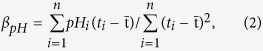


where *pH*_*i*_ and *t*_*i*_ are pH and *t*, respectively at *t* = *t*_*i*_, and 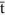 is the average *t*.

As *t* can only be used to model the linear temporal change in pH_insitu_, it cannot be used to model the strong seasonal variations. Suppose that other variables (e.g. *T*, dissolved oxygen, nutrients), *x*_*1*_*, …, x*_*n*_, are added to model the temporal variations in pH_insitu_, which can be expressed as follows,


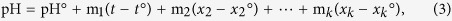


where m_1_ ~ m_*k*_ are constants, t° is a reference point of *t*, and 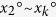 are *x*_2_ ~ *x*_*k*_ at *t* = t°, respectively.

Substituting Eq (3) into Eq (2), Eq (2) can be rewritten as follows,


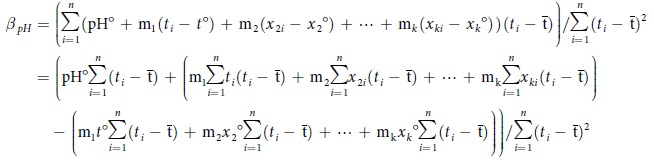


As 
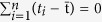
 and 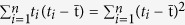, the equation above can be simplified as follows,


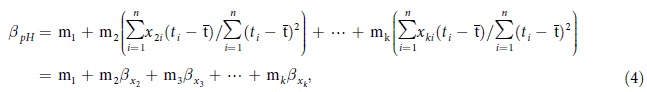


where 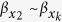
are SLR-determined rates of temporal changes in *x*_2_ ~ *x*_*k*_, respectively (see the definition shown in Eq (2)).

Equation (4) shows that in fact, *β*_*pH*_ can be expressed as a linear combination of a constant (m_1_) and rates of temporal changes in the other regression variables (
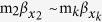
). Thus, although differences in the number of observation years and temporal distribution of sampling yield different *β*_*pH*_ and 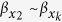, values for 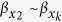 change with *β*_*pH*_, whereas m_1_ remains unchanged. Statistically, if *x*_2_ ~ *x*_*k*_ in the above equation have no long-term trends of changes, 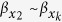 are expected to be zero. Non-zero 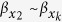 refers to the consequence of various sampling distributions under seasonal variations in *x*_2_ ~ *x*_*k*_. If the long-term trends of changes in *x*_2_ ~ *x*_*k*_ have no collinearities with that of pH_insitu_, any long-term trends of changes in *x*_2_ ~ *x*_*k*_ would not affect *β*_*pH*_.

Notably, m_1_ is defined as the amount of pH_insitu_ change as *t* changes. Therefore, if long-term changes in 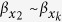 do not vary with that of *t* or pH_insitu_, m_1_ refers to the estimated rate of temporal change in pH_insitu_ due to different physical and biological forcings (e.g. changes in anthropogenic CO_2_ concentration, productivity, microbial respiration rate, etc.), whereas 
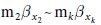
 refers to the deviation due to the temporal variation. Notably, the deviation due to uneven sampling distribution under strong temporal variations can be quantified using rates of temporal changes in other variables.

### Verifications and criteria of additional regression variables

To verify Eq (4), additional variables need to be added to the regression model shown in Eq (3). These variables must be strong in seasonal variations and weak in long-term changes, so that they can be used to model the seasonal variations in the pH_insitu_. At the same time they should have weak collinearities with *t*. Seawater *T* may serve this purpose, as pH_insitu_ is related to *T* and observations show that the *T* time series routinely mirrors the pH_insitu_ time series^6^,^7^. Furthermore, modelled results from recent studies show that the surface ocean pH_insitu_ has no observable change under a long-term change in *T*^15^,^16^.

To verify the appropriateness of using *t* and *T* in modeling pH_insitu_, Eq (3) is simplified as follows,


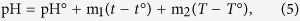


where T^o^ is *T* when *t* = t^o^.

Using the initial year of t° = 1988 as a reference, the multiple linear regression (MLR) model of Eq (5) is then applied to the surface ocean pH_insitu_ from five time series stations. The results are shown in Fig. 3 and Table 1.

### Linearity between pH_insitu_, *t*, and *T.*

Figure 3 shows the observed pH_insitu_ time series and the modeled results using MLR at various time series stations. The regression results match well with the annual, interannual, and long-term variations in observations. The m_1_ and m_2_ of −1.6 ± 0.1 × 10^−3^ pH unit yr^−1^ and −8.7 ± 1.4 × 10^−3^ pH unit °C^−1^, respectively, are similar among the time series stations. The R^2^ values are increased from 0.09 ~ 0.55 to 0.51 ~ 0.94, with all sites averaging 0.75 ± 0.17. The differences between the measured and the MLR results are at most approximately twice the pH calculation error of ±0.0062, based on the carbonate system using total alkalinity (TA) and dissolved inorganic carbon^17^, suggesting the validation of Eq (5). Consequently, Eq (4) can now be simplified as follows,


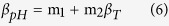


It is important to point out that the linearity between pH_insitu_ and *T* is a lump sum of various physical and biological forcings. Based on this linearity, the change in *β*_*pH*_ due to various sampling distributions under the strong seasonal variability is reflected in m_2_*β*_*T*_. The fact is that, changes in pH_insitu_ and *T* owing to other factors (e.g. global warming, enhanced upwelling, etc.) may not equal to m_2_, resulting in residuals. The standard errors of Eq (5) shown in Table 1 possibly reflect the errors from minor disturbances and from the measurements. Since Eq (6) is generated from Eq (5), Eq (6) contains the same kind of errors as Eq (5) does. Fortunately, the standard errors are relatively small. Therefore, m_1_ is the estimated rate of temporal change in pH_insitu_, and m_2_*β*_*T*_ is the deviation largely due to the various sampling distributions under the strong seasonal pH_insitu_ variability. In addition, any parameter, having linearity with pH_insitu_ and having no observable temporal collinearities with *t* or pH_insitu_, can technically be used in the same way as *T* does to quantify the deviation of *β*_*pH*_ due to various sampling distributions under strong seasonal pH_insitu_ variability.

### Linearity between *β*_*pH*_ vs. *β*_*T*_ and the deviation (m_2_ *β*_*T*_)

To quantify Eq (6), observations from five time series with varying numbers of observation years (9–23 years) are examined with the average *β*_*pH*_ of 31 time series stations along the 137 ^o^E line in the winter and summer (Fig. 4; detailed information are shown in Supplementary Fig. S1 Supplementary Information Table S1). Determined by SLR on the observed pH_insitu_ and *T* time series, the results show that the observed *β*_*pH*_ values vary from +1.61 × 10^−3^ pH unit yr^−1^ to −2.5 × 10^−3^ pH unit yr^−1^. The slope (m_2_) of the regression line of −9.7 ± 0.7 × 10^−3^ pH unit °C^−1^ is consistent with the MLR determined m_2_, with an average of −8.7 ± 1.4 × 10^−3^ pH unit °C^−1^ for the five time series (Table 1). Although no reported average values in m_1_ and m_2_ are given, the result in Fig. 4 shows that the average *β*_*pH*_ values at the 137 ^o^E likely have m_1_ and m_2_ values similar to that of the other five time series.

Assuming an air-sea CO_2_ equilibrium, the surface ocean has slight regionally distributed differences in acidification rates owing to differences in seawater carbonate chemistry^18^. Based on thermodynamic calculations using the average carbonate parameters at each station, the acidification rate among the five time series stations and the 137 ^o^E line are similar between −1.6 × 10^−3^ and −1.8 × 10^−3^ pH unit yr^−1^. As *β*_*pH*_ is negatively correlated with *β*_*T*_, the deviation (m_2_*β*_*T*_) is equal to zero when *β*_*T*_ is zero. Indeed, the regression line in Fig. 4 yields a *β*_*pH*_ of −1.5 ± 0.1 × 10^−3^ pH unit yr^−1^ at *β*_*T*_ = 0. This value agrees with the MLR-determined m_1_ of −1.6 ± 0.1 × 10^−3^ pH unit yr^−1^, and with the expected acidification rate of −1.6 × 10^−3^ ~ −1.8 × 10^−3^ pH unit yr^−1^, when considering only the observed global atmospheric pCO_2_ increments during the studied periods. Notably, the assumed, extreme case of uneven sampling distribution shown in Fig. 2 agrees well with the regression line (Fig. 4, red circle). As shown for the first time, and having the shortest observation years and the largest deviation from the expected acidification rate (positive *β*_*pH*_), the SEATS site is used as an example to illustrate the influence of sampling distribution on the *β*_*pH*_ in the following discussion. At the SEATS site, the observed *β*_*pH*_, m_2_, and *β*_T_ of the nine year study are 0.21 × 10^−3^ pH unit yr^−1^, −7.7 × 10^−3^ pH unit °C^−1^, and −0.23 °C yr^−1^, respectively (Fig. 4, Table 1). It is important to note that the estimated rate of temporal change in pH_insitu_ (m_1_) is *β*_*pH*_ – m_2_*β*_T_ = (0.21 × 10^−3^pH unit yr^−1^)−(−7.7 × 10^−3^ pH unit ^o^C^−1^) × (−0.23 °C yr^−1^) = −1.6 × 10^−3^ pH unit yr^−1^, which is statistically indistinguishable from the expected acidification rate, assuming the air-sea CO_2_ equilibrium.

## Discussion

The results above show that the *β*_*pH*_ among the studied time-series, at *β*_*T*_ = 0, reflects the increasing atmospheric CO_2_, while deviations from that value are mainly due to different deviations of m_2_*β*_*T*_ under various distributions of sampling points in the studied areas. In short, *β*_*pH*_ at ESTOC with only selected data (an extreme case of uneven sampling distribution), and at SEATS and CARIACO, deviates so much from the expected acidification rate due to increasing atmospheric CO_2_ because of the deviation indicated by the apparent decrease in the observed *T* at ESTOC and SEATS (positive deviation) and the apparent increase in *T* at CARIACO (negative deviation). The long-term time-series, such as BATS and HOT, have the *β*_*pH*_ statistically indistinguishable from the expected rate of −1.6 × 10^−3^ ~ −1.8 × 10^−3^ pH unit yr^−1^ assuming air-sea CO_2_ equilibrium. That is, although long-term time series may have evenly distributed datasets and negligible deviations in *β*_*pH*_, shorter time series may suffer large errors which are correctable by this method.

It has been suggested that the anomaly (observed pH_insitu_ or *T* minus climatology mean) be used to reduce seasonal variations, as it has been reported that *β*_*pH*_ is sensitive to these^2^. Comparing deseasoned *β*_*pH*_ from Bates, *et al.*^2^ and deseasoned *β*_*T*_ from Astor, *et al.*^9^, based on pH_insitu_ and *T* anomalies at the CARIACO site, the deseasoned *β*_*pH*_ is similar to the observed *β*_*pH*_ that contains a deviation of m_2_*β*_*T*_ (Fig. 4, green circle). The reason why the *β*_*pH*_ and the deseasoned *β*_*pH*_ contain similar deviations needs further investigation.

The observed slope of −9.7 ± 0.7 × 10^−3^ pH unit °C^−1^ shown in Fig. 4 can only be due to changing seawater chemistry, and not to deviations, if the following two conditions are satisfied. First, *β*_*T*_ is a fair estimation of changing *T*. Second, the change in pH_insitu_ under long-term change in *T* is equal to the m_2_ of −9.7 ± 0.7 × 10^−3^ pH unit °C^−1^. However, the second assumption contradicts with our thermodynamic calculation that the change in pH_insitu_ as *T* changes (m_2_) is in fact just approximately −1.1 × 10^−3^ pH unit ^o^C^−1^, which is based on the assumptions of air-sea CO_2_ equilibrium and constant TA. This is consistent with recent studies showing that the change in surface ocean pH_insitu_ under long-term change in *T* (m_2_) is close to zero^15^,^16^. Seasonal changes in *T* are much larger than long-term changes in *T*, such that the observed m_2_ of −8.7 ± 1.4 × 10^−3^ and −9.7 ± 0.7 × 10^−3^ pH unit °C^−1^ shown in Table 1 and Fig. 4, respectively, largely represents the values of seasonal change in pH_insitu_ as *T* changes. Notably, uneven sampling generates a deviation in *β*_*T*_ as well. Although deducing the deviation in *β*_*T*_ under uneven sampling distribution is beyond the scope of this study, this does not change a fact that based on Eq (6), in the case of uneven distribution of sampling the slope of *β*_*pH*_ vs. *β*_*T*_ plot should be approximately equal to m_2_ = −8.7 ± 1.4 × 10^−3^pH °C^−1^. In contrast, the slope of fair estimation is expected to be just about −1.1 × 10^−3^ pH unit °C^−1^. The factor of −9.7 ± 0.7 × 10^−3^ *β*_*T*_ shown in Fig. 4 is actually a deviation of the acidification rate largely owing to various distributions of sampling points, rather than an effect on the acidification rate due to warming or cooling.

In the marine carbonate system, the TA concentration should remain constant if the change in pH_insitu_ is solely caused by the air-sea CO_2_ exchange. To examine the temporal change in the TA concentration, it is normalized to a particular salinity (S), such as at S = 35 (nTA = TA/S×35), to reduce the influences of precipitation or evaporation on the TA concentration. At the ESTOC and the 137 °E line, the nTA concentrations have reportedly remained unchanged over the study regions and periods^8^,^12^. The rates of temporal change in nTA (*β*_*nTA*_) among the rest of the four sites are also found to be insignificant within the uncertainties of the data (see Supplementary Table S1 for detail). Further, changes in TA through precipitation or evaporation have insignificant influences on *β*_*pH*_ among those time series stations. For instance, at the HOT site, a change in TA as S changes (11 × 10^−3^ S unit yr^−1^) yields a *β*_*pH*_ of only 0.091 × 10^−3^ pH unit yr^−1^. Such a magnitude of change is less than 5% of the reported *β*_*pH*_ at the HOT site. In fact, the constant nTA among the studied time series leads to the conclusion that m_1_ of −1.6 × 10^−3^ pH unit yr^−1^ in Table 1 and −1.5 × 10^−3^ pH unit yr^−1^ in Fig. 4 indeed reflect the increasing atmospheric CO_2_ concentration.

To conclude, long-term time series with even sampling distribution contains negligible deviation. However, a short duration of observation years, low sampling frequency, and high seasonal variations in the pH_insitu_ time series tend to result in a temporal change with a large deviation. For instance, due to its high seasonal variation in pH_insitu_ and short duration, in the SEATS study, the temporal increase in pH_insitu_ due to uneven distributions of data is large enough to compensate for decreasing pH_insitu_ caused by increased atmospheric CO_2_. *T* is used as an example to quantify the deviation. Once the deviation is eliminated, seawaters at the studied stations are acidified at a rate statistically indistinguishable from the expected rate under the air-sea CO_2_ equilibrium. Our results indicate that the differences in *β*_*pH*_ among the studied time series are due to deviations using SLR. As our approach is not so limited by the number of observation years or the sampling distribution, time series studies with high seasonal variations and short duration times can benefit from our approach, achieving a fair estimation of the rate of temporal change in pH_insitu_.

## Methods

**Figure 1:**
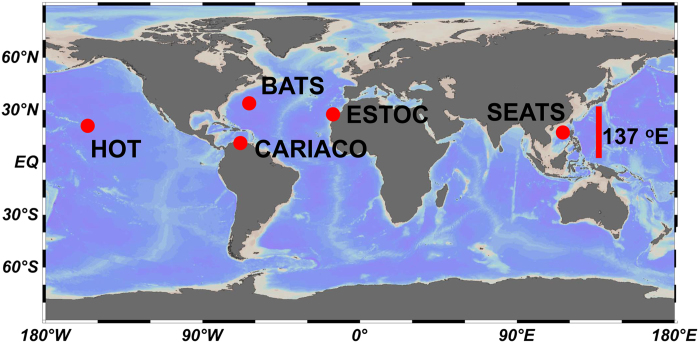
Station locations of the time series, including Bermuda Atlantic Time Series Study (BATS), Carbon Retention in a Colored Ocean Project (CARIACO), European Station for Time Series in the Ocean Canary Islands (ESTOC), Hawaii Ocean Time Series (HOT), South East Asia Time Series Study (SEATS), and 137°E repeated hydrographic line (137°E). The map was generated using Ocean Data View 4.6.2. Schlitzer, R., Ocean Data View, odv.awi.de, 2015.

**Figure 2:**
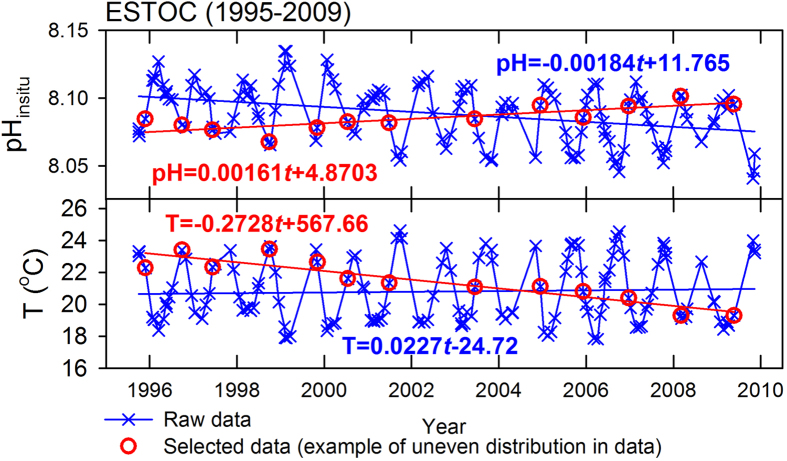
Time series of pH_insitu_ and *T* at ESTOC. The blue crosses and lines show the raw data and the regression lines. The red circles and lines show a case of selected data, and the regression lines. Data taken from Gonzalez-Davila and Santana-Casiano^19^.

**Figure 3:**
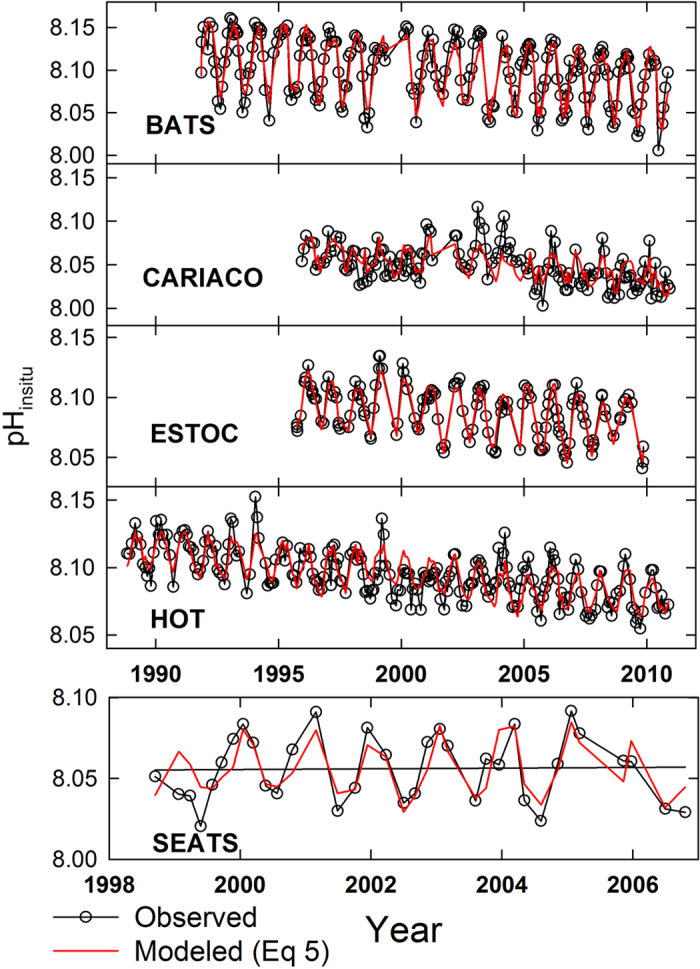
The surface seawater pH_insitu_ at various time series stations. The black and red lines show, respectively, the observed values and the values modeled by Eq (5).

**Figure 4:**
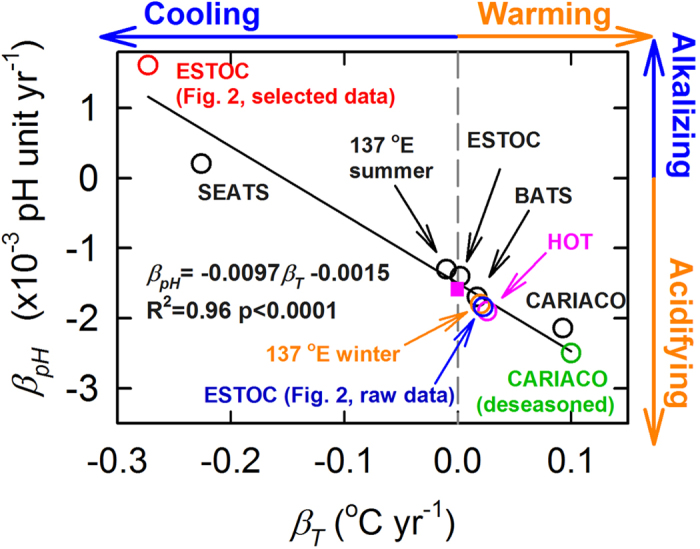
The observed surface seawater *β*_pH_ vs. *β*_T_ at various time series stations. The solid line shows the linear regression result. The pink solid square shows the expected acidification rate based on the assumption of air-sea CO_2_ equilibrium. The vertical dashed line represents zero *β*_*T*_. See the text for the names of the time series studies.

The data sets (see Fig. 1 for station locations) used in this study are from five published time series studies (the BATS^6^, CARIACO^2^,^9^, ESTOC^8^,^19^, HOT^7^, and the 137°E^12^) and an unpublished, open access pH time series study (the SEATS). In this study, the up-to-date CO_2_ System Calculations Program (version 2.1) developed by *Pierrot, et al.*^20^ and the recommended dissociate constants of carbonate chemistry for best practices^21^,^22^ were used to calculate the carbonate system. The pH is in the total scale at the *in situ* temperature. Numbers are expressed as the value ± one standard error.

## Additional Information

**How to cite this article**: Lui, H. K. and Chen, C. T. A. Deducing acidification rates based on short-term time series. *Sci. Rep.* **5**, 11517; doi: 10.1038/srep11517 (2015).

## Supplementary Material

Supplementary Information

## Figures and Tables

**Table 1:** MLR coefficients of Eq (5) of seawater pH_insitu_ from various time series studies. The pH° and T° are determined using the SLR method when t° = 1988 (see Supplementary Table S2).

		Eq (5) pH = pH° + m_1_(*t*−t°) + m_2_(*T*−T°)						
Station	Year	pH^O^	T°	m_1_ x10^−3^ pH unit yr^−1^	m_2_ x10^−3^ pH unit ^o^C^−1^	Standard Error	R^2^	n
BATS	1991–2010	8.1297	22.85	−1.8 ± 0.2	−9.9 ± 0.3	0.0135	0.86	215
CARIACO	1995–2010	8.0835	24.82	−1.5 ± 0.3	−7.0 ± 0.7	0.0155	0.51	144
ESTOC	1995–2009	8.1155	20.46	−1.6 ± 0.1	−9.6 ± 0.2	0.0053	0.94	144
HOT	1988–2010	8.1172	24.68	−1.7 ± 0.1	−9.3 ± 0.5	0.0088	0.77	222
SEATS	1998–2006	8.0531	30.63	−1.5 ± 0.9	−7.7 ± 1.0	0.0123	0.66	35
Average		8.0998 ± 0.0311	24.69 ± 3.76	−1.6 ± 0.1	−8.7 ± 1.4	0.011 ± 0.004	0.75 ± 0.17	5
