# Supplementary material for: Deducing acidification rates based on short-term time series
Source: Sci Rep. 2015 Jul 6;5:11517. doi: 10.1038/srep11517 (PMC4648385; doi:10.1038/srep11517)
Supplement: Supplementary Information [file srep11517-s1.pdf]

Supplementary Information

Deducing acidification rates based on short-term time series

Hon-Kit Lui and Chen-Tung Arthur Chen*

Department of Oceanography,

National Sun Yat-Sen University, Kaohsiung 80424, Taiwan.

*Corresponding author

Tel: +886-7-525-5136;

Email: ctchen@mail.nsysu.edu

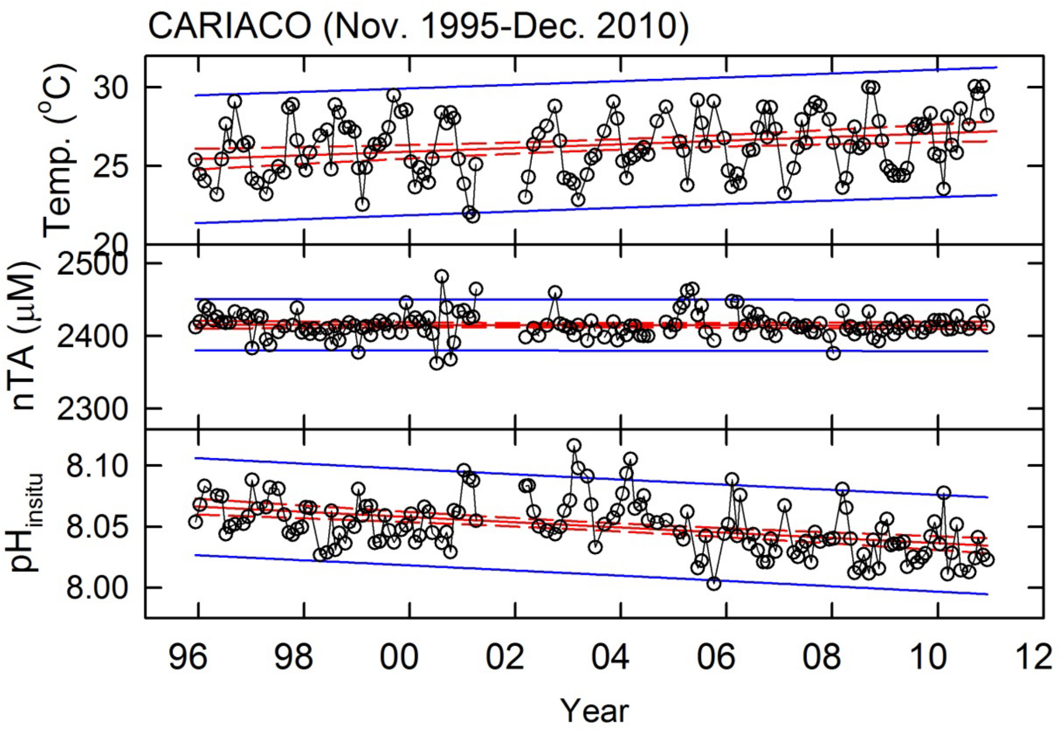

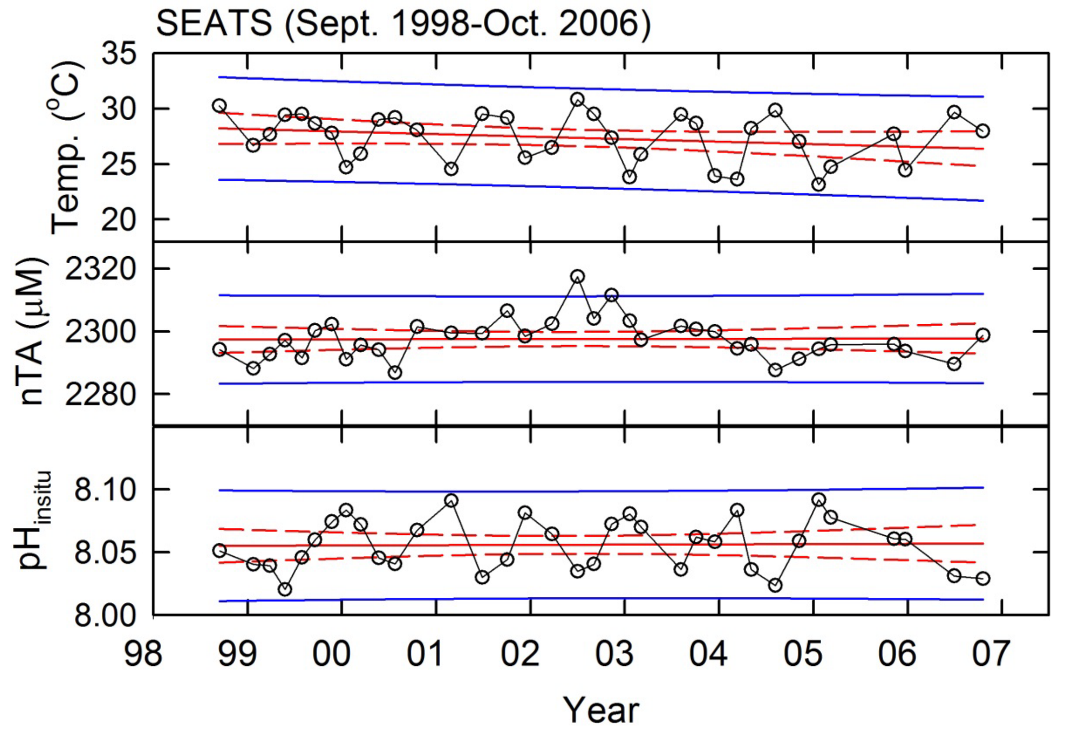

Supplementary Figure S1. Time series of temperature, normalized total alkalinity (nTA) and pH_{insitu} at CARIACO and SEATS. The red solid and dashed lines are the linear regressions and 95 % confidence intervals, respectively, while blue lines are the 95% prediction intervals of linear regressions. Rates of temporal changes in temperature, nTA and pH_{insitu} at CARIACO and SEATS are shown in Table S1.

Supplementary Table S1. The observed surface seawater β_{pH}, β_{T} and β_{nTA} at various time-series stations.

Time series study	Studied Period	β_{pH} ($\times 10^{-3}$ pH unit yr^{-1})	β_{T} (°C yr^{-1})	β_{nTA} (μmolkg^{-1} yr^{-1})
BATS[1]	1983-2005	-1.7±0.3	0.017±0.030	0.06±0.04
CARIACO[a] (Figure S1)	1995-2010	-2.14±0.37	0.093±0.034	-0.067±0.305
CARIACO[2]	1996-2008			0.1±0.33
CARIACO (deseasoned)	1995-2014	-2.5±0.4 [3]	0.1±0.04 (1996-2008)[2]	
ESTOC[4,5]	1995-2004	-1.4±0.7	0.002±0.019	Reported to have no observable change
ESTOC[b] (Figure 1, blue crosses)	1995-2009	-1.84±0.39	0.023±0.039	-0.028±0.039
ESTOC[b] (Figure 1, red circles)	1995-2009	1.61±0.37	-0.273±0.036	
HOT[6]	1988-2007	-1.9±0.2	0.026±0.016	-0.06±0.06
SEATS[c] (Figure S1)	1998-2006	0.206±1.50	-0.226±0.157	0.04±0.48
137 °E[7]	1983-2007	Winter: -1.8±0.2<br>Summer: -1.3±0.5	0.02±0.02<br>-0.01±0.02	Reported to have no observable change

[a]Data taken from the Institute of Marine Remote Sensing, College of Marine Science, University of South Florida. http://www.imars.usf.edu/CAR/

[b]Data taken from Gonzalez-Davila and Santana-Casiano [8].

[c]Data provided by the Ocean Data Bank (ODB), Ministry of Science and Technology, Taiwan.

Supplementary Table S2. SLR equations of the pH_{insitu} and T time series at various time-series stations. (Units: T is ^{o}C, t is year)

Station	Year	$pH = pH° + \beta_{pH}(t - 1988)$<br>$T = T° + \beta_T(t - 1988)$	Standard error	R^2	n
BATS	1991-	$pH = 8.1297 - 0.0022(t - 1988)$	0.034	0.122	215
	2010	$T = 22.85 + 0.033(t - 1988)$	3.10	0.004	
CARIACO	1995-	$pH = 8.0835 - 0.00214(t - 1988)$	0.020	0.194	144
	2010	$T = 24.82 + 0.093(t - 1988)$	1.86	0.049	
ESTOC	1995-	$pH = 8.1155 - 0.00184(t - 1988)$	0.020	0.135	144
	2009	$T = 20.46 + 0.023(t - 1988)$	1.96	0.002	
HOT	1988-	$pH = 8.1172 - 0.00183(t - 1988)$	0.014	0.399	222
	2010	$T = 24.68 + 0.015(t - 1988)$	1.20	0.006	
SEATS	1998-	$pH = 8.0531 + 0.000206(t - 1988)$	0.021	0.0006	35
	2006	$T = 30.63 - 0.226(t - 1988)$	2.17	0.059	

References:

1 Bates, N. R. Interannual variability of the oceanic CO_2 sink in the subtropical gyre of the North Atlantic Ocean over the last 2 decades. *J. Geophys. Res.-Oceans* **112**, doi:C09013 10.1029/2006jc003759 (2007).

2 Astor, Y. M. *et al.* Interannual variability in sea surface temperature and fCO_2 changes in the Cariaco Basin. *Deep-Sea Res. Part II-Top. Stud. Oceanogr.* **93**, 33-43, doi:10.1016/j.dsr2.2013.01.002 (2013).

3 Bates, N. R. *et al.* A time-series view of changing ocean chemistry due to ocean uptake of anthropogenic CO_2 and ocean acidification. *Oceanography* **27**, 126-141, doi:10.5670/oceanog.2014.16 (2014).

4 Gonzalez-Davila, M., Santana-Casiano, J. M., Rueda, M. J. & Llinas, O. The water column distribution of carbonate system variables at the ESTOC site from 1995 to 2004. *Biogeosciences* **7**, 3067-3081, doi:10.5194/bg-7-3067-2010 (2010).

5 Santana-Casiano, J. M., Gonzalez-Davila, M., Rueda, M. J., Llinas, O. & Gonzalez-Davila, E. F. The interannual variability of oceanic CO_2 parameters in the northeast Atlantic subtropical gyre at the ESTOC site. *Glob. Biogeochem. Cycle* **21**, doi:Gb101510.1029/2006gb002788 (2007).

6 Dore, J. E., Lukas, R., Sadler, D. W., Church, M. J. & Karl, D. M. Physical and biogeochemical modulation of ocean acidification in the central North Pacific. *Proc. Natl. Acad. Sci. U. S. A.* **106**, 12235-12240, doi:10.1073/pnas.0906044106 (2009).

7 Midorikawa, T. *et al.* Decreasing pH trend estimated from 25-yr time series of carbonate parameters in the western North Pacific. *Tellus Ser. B-Chem. Phys. Meteorol.* **62**, 649-659, doi:10.1111/j.1600-0889.2010.00474.x (2010).

8 Gonzalez-Davila, M. & Santana-Casiano, J. M. Sea Surface and Atmospheric fCO_2 data measured during the ESTOC Time Series cruises from 1995-2009. *http://cdiac.ornl.gov/ftp/oceans/ESTOC_data/. Carbon Dioxide Information Analysis Center, Oak Ridge National Laboratory, US Department of Energy, Oak Ridge, Tennessee.,* doi:10.3334/CDIAC/otg.TSM_ESTOC (2009).
